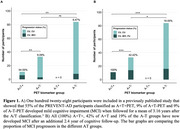# The clinical prognosis of cognitively unimpaired individuals with positive PET biomarkers

**DOI:** 10.1002/alz70856_096588

**Published:** 2025-12-24

**Authors:** Sylvia Villeneuve

**Affiliations:** ^1^ Douglas Mental Health University Institute, Centre for Studies on the Prevention of Alzheimer's Disease (StoP‐AD), Montréal, QC, Canada; McGill University, Montreal, QC, Canada; StoP‐AD Centre, Douglas Mental Health Institute Research Centre, Montreal, QC, Canada

## Abstract

It is a matter of debate if cognitively unimpaired (CU) individuals with Alzheimer pathology should be classified as having Alzheimer's disease,^1^ or if this terminology should be restricted to individuals with cognitive impairments.^2^ Central to this debate is the uncertainty that all individuals with Alzheimer pathology will develop major cognitive impairments if followed over time.

In 2022, two multi‐cohort studies showed that half of CU individuals identified as being abnormal on both amyloid (A+) and tau (T+) positron emission tomography (PET) biomarkers developed mild cognitive impairment (MCI) or dementia when followed for a mean of 3.5 years.^3,4^ Individuals only positive on the amyloid scan (A+T‐) were not at increased risk of developing MCI or dementia when compared to A‐T‐ individuals. We recently updated the cognitive status of participants enrolled in one of these studies and found that, with an additional 2.4 years of study follow‐up, 100% of the PREVENT‐AD participants who were A+T+ have now progressed to MCI or dementia (Figure 1). Of high importance, the percentage of progressors in the A+T‐ group increased from 9.09% to 42.42%.

These results provide clear evidence that CU older adults with both amyloid plaques and tau aggregates measured with PET will develop cognitive impairments if the disease is not stopped. The deterministic nature of the A+T‐ biomarker group is more ambiguous, but longer follow‐up clearly shows worse prognosis and individuals who are A+T‐ probably have some level of tau pathology even if not yet detectable by tau PET tracers and conventional positivity thresholds.

The goal of this presentation is to discuss the prognostic value of amyloid and tau PET biomarkers at identifying CU who will develop MCI in a 6‐year window. We will also discuss new approaches to identify amyloid and tau PET positivity that might be more sensitive than conventional visual read or quantitative binary thresholds. This knowledge is important for enrolling individuals in clinical trials given that some treatments might work best in individuals with low levels of pathology.

References

1. Jack, et al., 2024

2. Dubois et al., 2024

3. Strikwerda‐Brown, et al., 2022

4. Ossenkoppele, et al., 2022